# Simulation Study of Inertial Micro-Switch as Influenced by Squeeze-Film Damping and Applied Acceleration Load

**DOI:** 10.3390/mi7120237

**Published:** 2016-12-20

**Authors:** Yingchun Peng, Zhiyu Wen, Dongling Li, Zhengguo Shang

**Affiliations:** 1 Microsystem Research Center, Chongqing University, Chongqing 400044, China; wzy@cqu.edu.cn (Z.W.); lidongling@cqu.edu.cn (D.L.); zhengry@cqu.edu.cn (Z.S.); 2 Key Laboratory of Fundamental Science of Micro/Nano-Device and System Technology, Chongqing University, Chongqing 400044, China

**Keywords:** inertial switch, MEMS, squeeze-film damping, acceleration, contact bounce, contact time, response time, threshold acceleration

## Abstract

Squeeze-film damping and acceleration load are two major issues in the design of inertial micro-switches. In order to deeply and systematically study these two issues, this paper proposes a typical vertically-driven inertial micro-switch, wherein the air and electrode gaps were chosen to design the required damping ratio and threshold value, respectively. The switch was modeled by ANSYS Workbench, and the simulation program was optimized for computational accuracy and speed. Transient analysis was employed to investigate the relationship between the damping ratio, acceleration load, and the natural frequency, and the dynamic properties (including contact bounce, contact time, response time, and threshold acceleration) of the switch. The results can be used as a guide in the design of inertial micro-switches to meet various application requirements. For example, increasing the damping ratio can prolong the contact time of the switch activated by short acceleration duration or reduce the contact bounce of the switch activated by long acceleration duration; the threshold value is immune to variations in the damping effect and acceleration duration when the switch is quasi-statically operated; the anti-jamming capability of the switch can be improved by designing the sensing frequency of the switch to be higher than the acceleration duration but much lower than the other order frequencies of the switch.

## 1. Introduction

As a typical inertial device for acceleration sensing, the inertial switch has been extensively investigated over the last several decades. It works as both a sensor and an actuator, and is normally open until the acceleration event occurs, significantly simplifying the complexity of the system and reducing the power requirement. Recently, with the advantages of small size, high integration level, and low cost [1,2,3,4], the micro-electro-mechanical-system (MEMS) technology-based inertial micro-switch is widely available in large-volume commercial applications, such as airbags, transportation systems, and geriatric healthcare [5,6]. The working principle of the inertial micro-switch is that a movable electrode (usually a proof mass suspended by compliant springs) contacts a fixed electrode when an acceleration exceeding the threshold value is applied, consequently shorting the external circuit. The contact process is complicated, and the dynamic properties, such as contact bounce, contact time, response time, and threshold acceleration are essential factors to determine the feasibility of practical applications. As such, it is meaningful to investigate the influences on the dynamics of the inertial micro-switch.

The squeeze-film damping effect for a vertically-driven switch to detect out-of-plane acceleration has recently been a major issue in the design of inertial micro-switches. Michaelis et al. [7] designed the squeeze-film damping ratio of their device to be 0.7 to enable a constant threshold detection function that is independent of the acceleration pulse length. Matsunaga and Esashi [8] designed their switch to maximize the squeeze-film damping effect on the proof mass to extend the time of the on-state of the switch. Younis et al. [9] indicated that the squeeze-film damping effect becomes a major factor when the switch is excited dynamically, while the effect is significantly weakened in the case of quasi-static impact load. Currano et al. [10] demonstrated that the response time of the switch and the acceleration level required to turn the switch on increase as the associated damping factor is increased. Dehkordi et al. [11] adjusted the squeeze-film damping effect to reduce the response delay of the switch and to increase the contact time.

Another important factor influencing the dynamics of the inertial micro-switch is the applied acceleration load, mainly including the amplitude and duration (pulse width). McNamara and Gianchandani [12] demonstrated that the switch cannot be triggered if the acceleration duration is sufficiently short. Younis et al. [9] designed their switch to respond quasi-statically to mechanical shock, making the switch robust against variations in shock shape and duration. Moreover, Younis et al. [9] indicated that quasi-static operation can make a switch insensitive to variations in damping conditions. Yang et al. [13] claimed that the contact time of the inertial micro-switch is very short (usually less than 10 μs) when the switch is excited dynamically, while the contact bounce becomes the main problem in the case of quasi-static operation. Yang et al. [13] designed a micro-switch as a dual mass-spring system (both the proof mass and the contact point on it are suspended by springs) to reduce the bouncing effect and prolong the contact time. Kim et al. [14] suggested that the resonant frequency of the device should be located higher than the duration of input waveform to minimize the threshold discrepancy between steady state and half-sinusoidal input. Chen et al. [15] studied the influence of applied acceleration load on contact time and threshold acceleration in an inertial micro-switch and concluded that the contact time increases with the increase of acceleration amplitude and the broadening of acceleration duration, and the threshold acceleration increases with the broadening of acceleration duration.

From the above-mentioned review, we note that squeeze-film damping and applied acceleration load have been the concern of many researchers in the design of inertial micro-switches. However, there are few research reports on the systematic study of the relationship between the squeeze-film damping, acceleration load, and the dynamics of the inertial micro-switch. In this paper, we propose an inertial micro-switch of typical vertically-driven structure, wherein the required damping ratio and threshold value can be separately determined by the air and electrode gaps, facilitating the single factor comparison of different dynamic responses of the switch under various damping ratios and acceleration loads. Transient analysis of ANSYS Workbench is employed to investigate the relationship between the damping ratio, acceleration load, and the natural frequency, and the dynamic properties (including contact bounce, contact time, response time, and threshold acceleration) of the switch. The results can provide guidance for future research into the design of inertial micro-switches.

## 2. Device Design

**Figure 1:**
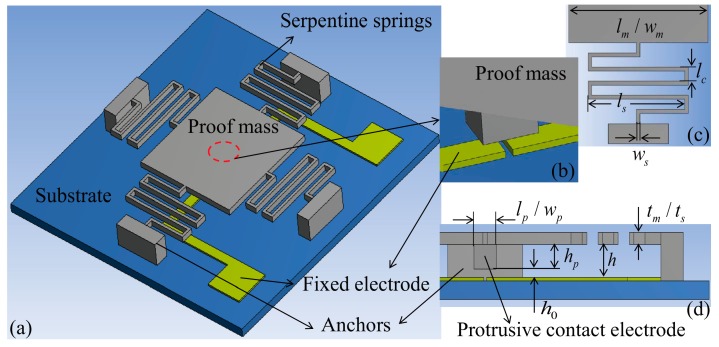
(**a**,**b**) Three-dimensional sketch and (**c**,**d**) main geometric specifications of the micro-switch.

The designed inertial micro-switch consists of a proof mass suspended by four serpentine springs as the sensitive element to detect out-of-plane acceleration, a protrusion positioned at the bottom center of the proof mass as the moveable contact electrode, two separated metal strips on the glass substrate as the double-contact-configuration fixed electrode. A three-dimensional sketch of the switch is shown in Figure 1a,b. When an acceleration exceeding the preset threshold level is applied to the switch in the sensitive direction, the proof mass deflects and traverses the electrode gap ($h_{0}$), making the moveable contact electrode short the fixed electrode and turning the switch on. The main geometric specifications and parameters are shown in Figure 1c,d and Table 1. The objective threshold acceleration was set at 15 *g* for low *g* acceleration applications such as missile health monitoring [5]. The values of h and $h_{0}$ were set as variables to design the desired damping ratio and threshold acceleration, respectively. The design strategy is described as follows.

As a typical inertial sensor, an inertial switch can be modeled by a mass-spring-damping system to represent the dynamics of the device. Considering that the acceleration applied to the device in practical work is a half-sine wave $a_{peak}\sin(\frac{\pi }{T_{0}}t)$, the under-damped (damping ratio ζ<1) response of the switch to the acceleration is [16]

(1) $$z{\left(t\right)}_{underdamped}=-a_{peak}\frac{\pi }{T_{0}}\left(\begin{array}{l} o\cos\left(\frac{\pi }{T_{0}}t\right)+\frac{pT_{0}}{\pi }\sin\left(\frac{\pi }{T_{0}}t\right)\\ +e^{-{\zeta \omega }_{n}t}\left(-o\cos\left(\omega_{d}t\right)+\frac{q+o{\zeta \omega }_{n}}{\omega_{d}}\sin\left(\omega_{d}t\right)\right) \end{array}\right)\sin\left(\frac{\pi }{T_{0}}t\right),$$

where z(t) is the displacement of the proof mass relative to the substrate, $\omega_{n}$ is the natural frequency of the system, and:

$$a=\pi^{2}/{T_{0}}^{2},\;b=2{\zeta \omega }_{n},\;d={\omega_{n}}^{2},\;\omega_{d}=\omega_{n}\sqrt{1-\zeta^{2}},$$

$$o=\frac{-b}{{\left(a-d\right)}^{2}+ab^{2}},\;p=\frac{d-a}{{\left(a-d\right)}^{2}+ab^{2}},\;q=\frac{a+b^{2}-d}{{\left(a-d\right)}^{2}+ab^{2}}.$$

The over-damped (damping ratio ζ> 1) response of the switch to the acceleration is [16]

(2) $$z{\left(t\right)}_{overdamped}=-a_{peak}\frac{\pi }{T_{0}}\left(\begin{array}{l} o\cos\left(\frac{\pi }{T_{0}}t\right)+\frac{pT_{0}}{\pi }\sin\left(\frac{\pi }{T_{0}}t\right)\\ +Je^{-\left(\zeta -\sqrt{\zeta^{2}-1}\right)\omega_{n}t}+Ke^{-\left(\zeta +\sqrt{\zeta^{2}-1}\right)\omega_{n}t} \end{array}\right)\sin\left(\frac{\pi }{T_{0}}t\right),$$

where $J=\frac{o\omega_{n}\left(\zeta -\sqrt{\zeta^{2}-1}\right)+q}{2\omega_{n}\sqrt{\zeta^{2}-1}}$, $K=-\frac{o\omega_{n}\left(\zeta +\sqrt{\zeta^{2}-1}\right)+q}{2\omega_{n}\sqrt{\zeta^{2}-1}}$.

Based on Equations (1) and (2), the required threshold acceleration $a_{th}$ (i.e., $a_{peak}$) can be obtained by designing the value of $h_{0}$ (i.e., ${\left|z\left(t\right)\right|}_{\max}$). In this structural design, the value of $h_{0}$ is adjusted by changing the height of the protrusion ($h_{p}$) when the air gap between the proof mass and substrate (h) is fixed for designing the desired squeeze-film damping ratio.

The squeeze-film damping effect is caused by the air surrounding the structure when the proof mass moves toward the substrate. The measure for the fluid continuity is the Knudsen number, $K_{n}=\lambda /w$, where λ≈0.1 μm is the mean free path of the air under 1 atm (standard atmospheric pressure) [17], and w is the initial air gap.

For the gas film between the proof mass and substrate, the gap height w=h≥35 μm. As such, we have $K_{n}<0.01$, and thus the fluid is in the continuum regime [18]. The behavior of the continuous fluid is generally governed by the well-known Reynolds equation [19]. Assuming that the gas is incompressible and the displacement of the proof mass is small, the Reynolds equation can be linearized as [20]:

(3) $$\frac{\partial^{2}P}{\partial x^{2}}+\frac{\partial^{2}P}{\partial y^{2}}=\frac{12\mu }{h^{3}}\frac{dh_{0}}{dt},$$

where $P=P_{0}-P_{a}$, $P_{0}$ is the pressure in the gas film, $P_{a}$ is the ambient pressure, x and y are the coordinate axes of the plane in parallel with the proof mass, $\mu =1.81\;\times \;10^{-5}\;\mathrm{Pa}\cdot s$ is the fluid viscosity of the air under 1 atm and 20 °C [17], and $h_{0}$ is the displacement of the proof mass. The temperature variation of the gas film is also ignored due to the small device size. The gas compressibility is evaluated by the squeeze number [20]:

(4) $$\sigma =\frac{12\mu \omega l^{2}}{P_{a}h^{2}},$$

where ω and l are the oscillating radial frequency and typical length of the proof mass, respectively. The gas can be considered as incompressible when σ is much smaller than unity, which is less than 0.06 in this paper. The requirement of small amplitude displacement of the proof mass is that $h_{0}$ is much smaller than h, which is met in this paper because $h_{0}$ is only several micrometers.

Based on Equation (3), the expression of the squeeze-film damping viscous coefficient for the oscillating proof mass is [20]

(5) $$c=\frac{\mu l_{m}{w_{m}}^{3}}{h^{3}}\beta \left(w_{m}/l_{m}\right),$$

where $l_{m}$ and $w_{m}$ are the length and width of the proof mass, respectively, and $\beta \left(w_{m}/l_{m}\right)$ is a correction factor and is equal to 0.42 when $w_{m}=\;l_{m}$. As such, according to the damping ratio expression of $\zeta =c/2m\omega_{n}$, where m is the mass of the proof mass, the required ζ can be obtained by designing the value of h.

For the gas film between the protrusion and substrate, the gap height $w=h_{0}<10\;\mu m$. Thus, we have $K_{n}>0.01$, and the fluid rarefaction effect [21] of the gas should be taken into account. Moreover, the displacement of the protrusion is equal to the air gap, and the large amplitude effect [22] should be considered. By substituting the standard fluid viscosity (μ) with an effective term [23,24,25,26] and increasing the damping viscous coefficient by a factor [22], however, it was found that the corresponding damping ratio is on the order of 0.001, and is thus ignored. This is due to the small size of the protrusion.

The analyses above indicate that the threshold acceleration and squeeze-film damping ratio can be separately determined by the electrode and air gaps (i.e., $h_{0}$ and h). Furthermore, the changing of $h_{0}$ (or $h_{p}$ as mentioned above) or h has no effect on the main mechanical properties of the device, such as m (the mass of the protrusion is neglected) and $\omega_{n}$. Therefore, the single-factor comparison of different dynamic responses of the switch under various damping ratios and acceleration loads is feasible.

## 3. Results and Discussion

**Figure 2:**
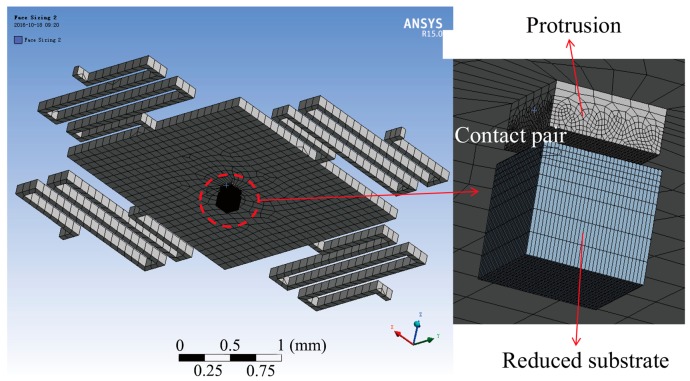
Finite element model of the micro-switch with the contact pair of the protrusion and reduced substrate.

The micro-switch was modeled by ANSYS Workbench software to simulate its dynamic response, as shown in Figure 2. The protrusion and substrate were defined as the contact and target bodies, respectively, to analyze the contact behavior between the two electrodes, and the contact type was frictionless-solid. The end sections of the four suspended springs and the back side of the substrate were constrained to be zero in all degrees of freedom. Transient analysis was employed to obtain the displacement–time curves of the protrusion under various squeeze-film damping ratios and acceleration loads. The time integration method of Hilber-Hughes-Taylor (HHT)-α was used to improve the stability and accuracy of the results. Due to the deformation (even slight) in the interface of the protrusion and substrate during the contact process, the simulation can be time-consuming. As such, in order to guarantee the computational accuracy and improve the computational speed, several optimization steps were implemented: first, the area of the substrate was reduced to only slightly larger than that of the protrusion; second, the model was firstly roughly meshed by the method of Hex Dominant, and then the contact area was meshed finer (element size =5 μm); third, the total step number of the load was set at 30, and the numbers of the substep (including initial substep, minimum substep and maximum substep) were set by two schemes—2, 1, and 3, respectively, for the case when the two electrodes were separated from each other, and 10, 6, and 12, respectively, for the case when the two electrodes were in contact with each other. The step end time of the analysis settings was set according to the acceleration duration. Based on the three steps above, the time required for each simulation was only several minutes. In contrast, the time required for the simulation of the same computational accuracy can be longer than ten hours when the second and third optimization steps are not carried out. The main material properties of the device structure used in this simulation are shown in Table 2.

**Figure 3:**
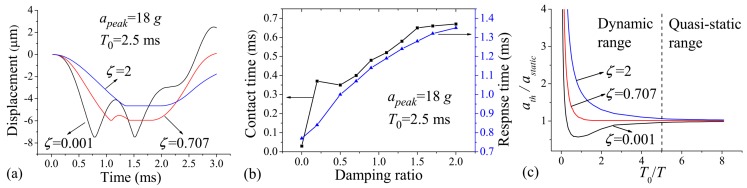
(**a**) Displacement vs. time when $a_{peak}=18\;g$, $T_{0}=2.5\;\mathrm{ms}$, and ζ=0.001, 0.7, and 2, respectively; (**b**) Contact time vs. damping ratio and response time vs. damping ratio when $a_{peak}=18\;g$ and $T_{0}=2.5\;\mathrm{ms}$; (**c**) $a_{th}/a_{static}$ vs. $T_{0}/T$ when ζ=0.001, 0.7, and 2, respectively.

Figure 3a shows the displacement–time curve of the protrusion for cases when the damping ratio ζ=0.001 (h=423 μm), 0.707 (h=49.2 μm), and 2 (h=35 μm). ζ=0.001 is assumed for the case that the squeeze-film damping effect can be neglected; ζ=0.707 is the most representative value employed by many researchers in this field, and ζ=2 is used to provide great enough damping effect. The applied acceleration duration, $T_{0}$, was 2.5 ms. The applied acceleration amplitude $a_{peak}=18\;g$ is 20% overload value of $a_{th}$ to make sufficient contact behavior. As seen in Figure 3a, the movement of the protrusion was stopped by the substrate, and then a period of stable contact (contact time) or frequent contact (contact bounce) occurred. Note from the figure that the contact bounce was evidently strong, and the contact time was very short when ζ=0.001; the amplitude of the contact bounce was significantly decreased, and the contact time was prolonged in the case of ζ=0.707; the contact bounce was finally eliminated, and the contact time was further prolonged when ζ=2. The explanation of the results is as follows: the rebound motion of the proof mass is constrained by the squeeze-film damping, and then the protrusion can be held near the substrate under the continuous action of the acceleration. The response time—defined as the time needed for the protrusion to reach the substrate—can also be delayed by the squeeze-film constraint effect. From Figure 3a, it can be seen that the response time is increased with increasing damping ratio. The effects of the squeeze-film damping on the contact time and response time were further investigated in Figure 3b. It can be seen that the contact time increases sharply when ζ increases from 0.001 to 0.2, and then increases relatively slowly when ζ is further increased to 2; the response time increases with the increasing of the damping ratio ranging from 0.001 to 2. The contact time can be up to about 650 μs when ζ=2, which is much longer than most of the values that have been published (the contact time is about 150–300 μs when the moveable and/or fixed electrode is designed elastically [4,27,28,29]).

In order to investigate the influences of the squeeze-film damping effect and acceleration duration on the threshold acceleration, we refer to the so-called shock-response spectrum analysis presented by Thomson [30] and further expand it for three damping ratios, ζ=0.001, 0.707, and 2, as shown in Figure 3c. The results are shown for the threshold acceleration of the micro-switch as a function of the ratio between $T_{0}$ to the natural period of the structure T. The threshold acceleration $a_{th}$ is normalized by the static threshold acceleration $a_{static}$ (the threshold value of the switch activated by sufficiently long acceleration duration). The plot is divided into two ranges: the dynamic load range when $T_{0}/T<5$, and the quasi-static load range when $T_{0}/T>5$. Figure 3c indicates that the threshold acceleration increases along with the damping ratio; the threshold acceleration is sensitive to variations in damping ratio and acceleration duration when the switch is excited dynamically; the threshold acceleration has less sensitivity to the damping ratio and acceleration duration in the case of quasi-static acceleration load. From Figure 3c, it can be seen that there can be a great discrepancy between $a_{static}$ and $a_{th}$ when $T_{0}$ is significantly smaller than T. In this case, the switch can be sensitive to the undesirable acceleration load of low frequency. As such, in order to minimize the discrepancy above, we recommend that the frequency of the switch should be designed to be higher than the duration of input acceleration. As seen in Figure 3c, for the normal value of $T_{0}/T>1$, the threshold acceleration (1) increases with increasing $T_{0}/T$ when ζ<0.707, while (2) decreases with increasing $T_{0}/T$ in the case of ζ>0.707. The conclusion of (1) is in agreement with that presented by Chen et al. [15], wherein the device damping ratio induced by slide-film damping is very small and thus can be neglected.

The mechanism of the quasi-static operation of the micro-switch can be simply explained as follows. For a large value of $T_{0}/T$ ($T_{0}/T>5$), the velocity of the proof mass moving toward the substrate is relatively low, and then the squeeze-film damping effect (which is proportional to the velocity) can be neglected. In this case, Equation (1) can be simplified as $z(t)=-\frac{a_{peak}}{{\omega_{n}}^{2}}\sin(\frac{\pi }{T_{0}}t)$ ($t<T_{0}$). It can be seen that the displacement of the proof mass closely tracks the applied acceleration load, and the threshold acceleration is independent of the damping effect and acceleration duration. Since microstructures with high natural frequency (i.e., small value of T) can be easily fabricated using MEMS technology (e.g., microstructure with short and/or thick beam and small proof mass), designing the switch to respond quasi-statically for most of the expected acceleration conditions is feasible.

**Figure 4:**
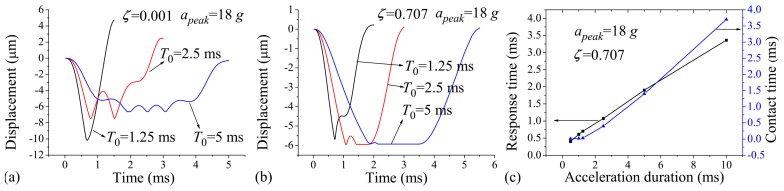
Displacement vs. time for (**a**) ζ=0.001 and (**b**) ζ=0.707 when $a_{peak}=18\;g$, $T_{0}=1.25\;\mathrm{ms}$, 2.5 ms, and 5 ms, respectively; (**c**) Contact time vs. acceleration duration and response time vs. acceleration duration when $a_{peak}=18\;g$ and ζ=0.707.

Figure 4a,b show the displacement–time curves of the protrusion under different acceleration durations of $T_{0}=1.25\;\mathrm{ms}$, 2.5 ms, and 5 ms. As mentioned above, the acceleration duration was set to be longer than that of the switch ca. 1.24 ms. The results are drawn for acceleration amplitude $a_{peak}=18\;g$ and damping ratios ζ=0.001 and 0.707. Figure 4a indicates that without the effect of the squeeze-film damping, the contact time can hardly be improved by increasing the acceleration duration, which will cause even more severe contact bounce. This is because without the effect of the squeeze-film damping, the rebound motion of the proof mass cannot be constrained, resulting in short contact time, and the number of bounce events may increase under the continuous action of the acceleration. These results show good agreement with the study presented by Yang et al. [13], who found that the contact bounce becomes serious when the switch is excited by quasi-static load. When ζ=0.707, however, the contact time can be prolonged with increasing acceleration duration, as shown in Figure 4b. From Figure 4a,b, it was found that the response time increases with the acceleration duration. The relationship between the contact time, response time, and the acceleration duration was further investigated for the cases when $T_{0}=0.5\sim 10$ ms and ζ=0.707, as shown in Figure 4c. The figure indicates that the contact time and response time generally increase with the broadening of the acceleration duration. The contact time is very short (ca. 10 μs) when $T_{0}=0.5\;\mathrm{ms}$, and simulation results show that the contact time is further shortened when $T_{0}<0.5\;\mathrm{ms}$. The transient contact time can make signal processing difficult for the external circuit on which the switch is integrated. As such, it is a great challenge for the application of inertial switch activated by acceleration load shorter than 0.5 ms.

**Figure 5:**
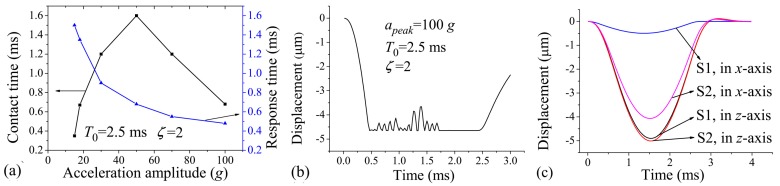
(**a**) Contact time vs. acceleration amplitude and response time vs. acceleration amplitude when $T_{0}=2.5\;\mathrm{ms}$ and ζ=2; (**b**) Displacement vs. time when $a_{peak}=100\;g$, $T_{0}=2.5\;\mathrm{ms}$, and ζ=2; (**c**) Displacement responses of two kinds of switches (S1 and S2) to the accelerations of 15 *g* and 2.5 ms applied in *x*- and *z*-axes.

Figure 5a shows the relationship between the contact time, response time, and the acceleration amplitude. It can be seen that the contact time first increases and then decreases with increasing acceleration amplitude, and the response time decreases with the acceleration amplitude. However, large acceleration amplitude may cause severe contact bounce, as shown in Figure 5b. Since the contact bounce may damage the interface of the two electrodes by mechanical hammering and electrical arcing [31], the micro-switch can fail when frequently suffering large acceleration amplitude.

The dynamics of the switch can also be influenced by the applied direction of acceleration load. For example, the components of the acceleration in insensitive directions will make the protrusion slide in the substrate plane, thus reducing the contact effect between the protrusion and substrate. This issue is usually evaluated by the anti-jamming capability of the switch. Figure 5c compares the displacement responses of two switches (named S1 and S2) to the applied acceleration loads of 15 *g* and 2.5 ms in the *z*-(sensitive direction) and *x*-(insensitive direction) axes. S1 and S2 are distinct in their spring widths (30 μm and 14 μm, respectively) and thicknesses (20 μm and 30 μm, respectively). By this design, S1 and S2 have essentially the same first-order frequency (808 Hz and 795 Hz in *z*-axis, respectively), and have almost the same displacement response in the *z*-axis (Figure 5c). However, the second-order frequency (1502 Hz in *x*- or *y*-axis) of S1 is much higher than the first one, but the second-order frequency (863 Hz in the *x*- or *y*-axis) of S2 is close to the first one. As such, the *x*-axis displacement of S1 is one order of magnitude smaller than that in the *z*-axis, and the two displacements are comparable in the case of S2 (Figure 5c). The results show that S1 has much better anti-jamming capability than S2. As such, in order to improve the anti-jamming capability of the switch, it is preferred to design the natural frequency of the switch in the sensitive direction much lower than those in insensitive directions.

Based on the simulation results above, we suggest that for micro-switches with rigid contact electrodes, the contact time can be prolonged by increasing the damping effect; for the application of short applied acceleration duration (e.g., the side airbag of a typical sedan ($T_{0}<5\;\mathrm{ms}$) [8]), it is preferred to design the damping ratio to be 0.7 to obtain acceptable contact time and reduce the sensitivity of the threshold acceleration to variation in acceleration duration; for the applications of long applied acceleration duration (e.g., the frontal airbag of a typical sedan ($T_{0}\approx 20\;\mathrm{ms}$) [8]), designing the micro-switch to be operated quasi-statically and with high damping ratio can achieve constant threshold value and stable contact effect.

## 4. Conclusions

In order to systematically investigate the effects of the squeeze-film damping and applied acceleration load on the dynamics of the inertial micro-switch, an inertial micro-switch of typical vertically-driven structure with a protrusion contact electrode positioned at the bottom center of the proof mass has been proposed in this paper. The required squeeze-film damping ratio and threshold value were separately determined by the air and electrode gaps, facilitating the single factor comparison of different dynamic responses of the switch under various damping ratios and acceleration loads. Finite-element simulation was employed to investigate the relationship between the damping ratio, acceleration load, and the natural frequency, and dynamic properties (including contact bounce, contact time, response time and threshold acceleration) of the switch. The results show that: first, increasing the squeeze-film damping effect can reduce the contact bounce and prolong the contact time, but will result in longer response time and higher threshold acceleration; second, broadening the acceleration duration can (1) prolong the contact time when the damping ratio is relatively high (e.g., ζ>0.2); (2) delay the response time; and (3) increase the threshold acceleration when ζ<0.707 while decreasing the threshold acceleration when ζ>0.707; third, the threshold acceleration is sensitive to variations in damping ratio and acceleration duration when the switch is excited dynamically, while it has less sensitivity to the damping ratio and acceleration duration in the case of quasi-static load; fourth, increasing the acceleration amplitude can prolong the contact time and shorten the response time, but may cause severe contact bounce; fifth, designing the natural frequency of the switch (in the sensitive direction) higher than the acceleration duration but much lower than those in insensitive directions can significantly improve the anti-jamming capability of the switch. The contact bounce was completely eliminated, and the contact time was up to ca. 650 μs when the damping ratio was set to 2. The conclusions obtained in this study provide a helpful reference for the design of inertial micro-switches.

## Figures and Tables

**Table 1:** Main geometric parameters of the micro-switch (μm).

$l_{m}/w_{m}$	$l_{p}/w_{p}$	$t_{m}/t_{s}$	$l_{s}$	$l_{c}$	$w_{s}$	h	$h_{0}$	$h_{p}$
2300	50	20	1600	150	30	Variable	Variable	$h-h_{0}$

**Table 2:** Main material properties of the device structure.

Material	Density	Young’s Modulus	Poisson’s Ratio
Silicon	2330 kg/m^3^	169 GPa	0.28
Glass	2200 kg/m^3^	70 GPa	0.17
